# Babesiosis as a potential threat for bovine production in China

**DOI:** 10.1186/s13071-021-04948-3

**Published:** 2021-09-07

**Authors:** Lan He, Reginaldo G. Bastos, Yali Sun, Guohua Hua, Guiquan Guan, Junlong Zhao, Carlos E. Suarez

**Affiliations:** 1 State Key Laboratory of Agricultural Microbiology, College of Veterinary Medicine, Huazhong Agricultural University, Wuhan, 430070 Hubei China; 2 Department of Veterinary Microbiology and Pathology, College of Veterinary Medicine, Washington State University, Pullman, WA 99164 USA; 3 State Key Laboratory of Plateau Ecology and Agriculture, College of Agriculture and Animal Husbandry, Qinghai University, Xining, 810016 People’s Republic of China; 4 Key Lab of Agricultural Animal Genetics, Breeding and Reproduction of Ministry of Education, International Joint Research Centre for Animal Genetics, Breeding and Reproduction, College of Animal Science & Technology, Huazhong Agriculture University, Wuhan, Hubei China; 5 State Key Laboratory of Veterinary Etiological Biology, Key Laboratory of Veterinary Parasitology of Gansu Province, Lanzhou Veterinary Research Institute, Chinese Academy of Agricultural Sciences, Xujiaping, Lanzhou, 730046 China; 6 Animal Disease Research Unit, United States Department of Agricultural - Agricultural Research Service, Pullman, WA 99164 USA

**Keywords:** Apicomplexa, *Babesia* spp., Bovine babesiosis, Tick-borne diseases, P. R. China, Chinese cattle industry

## Abstract

Babesiosis is a tick-borne disease with global impact caused by parasites of the phylum Apicomplexa, genus *Babesia.* Typically, acute bovine babesiosis (BB) is characterized by fever, anemia, hemoglobinuria, and high mortality. Surviving animals remain persistently infected and become reservoirs for parasite transmission. Bovids in China can be infected by one or more *Babesia* species endemic to the country, including *B. bovis*, *B. bigemina*, *B. orientalis*, *B. ovata*, *B. major*, *B. motasi*, *B.* U sp. Kashi and *B. venatorum*. The latter may pose a zoonotic risk. Occurrence of this wide diversity of *Babesia* species in China may be due to a combination of favorable ecological factors, such as the presence of multiple tick vectors, including *Rhipicephalus* and *Hyalomma*, the coexistence of susceptible bovid species, such as domestic cattle, yaks, and water buffalo, and the lack of efficient measures of tick control. BB is currently widespread in several regions of the country and a limiting factor for cattle production. While some areas appear to have enzootic stability, others have considerable cattle mortality. Research is needed to devise solutions to the challenges posed by uncontrolled BB. Critical research gaps include risk assessment for cattle residing in endemic areas, understanding factors involved in endemic stability, evaluation of parasite diversity and pathogenicity of regional *Babesia* species, and estimation of whether and how BB should be controlled in China. Research should allow the design of comprehensive interventions to improve cattle production, diminish the risk of human infections, and increase the availability of affordable animal protein for human consumption in China and worldwide. In this review, we describe the current state of BB with reference to the diversity of hosts, vectors, and parasite species in China. We also discuss the unique risks and knowledge gaps that should be taken into consideration for future *Babesia* research and control strategies.

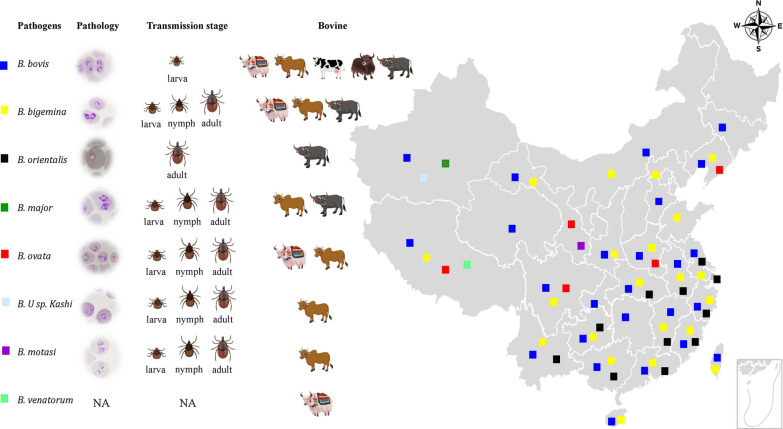

## Background

Babesiosis is a public health threat to human, domestic, and wild animals worldwide, especially in tropical and subtropical regions. The disease is caused by tick-borne apicomplexan parasites of the genus *Babesia* that invade erythrocytes in their vertebrate hosts. Bovine babesiosis (BB) is characterized by acute fulminant disease, typically in naïve cattle older than one year, and may result in either death or the development of persistent infection in animals that have recovered from the acute phase. Persistently infected animals become reservoirs for transmission by competent ticks. BB was first described in 1888 in Romania by Victor Babes as a disease caused by microorganisms residing inside of erythrocytes of cattle with hemoglobinuria [1]. At the time, the causative pathogen was initially named *Haematococcus bovis*, and later renamed *Babesia bovis* [2]. Since then, more than 100 *Babesia* species have been identified, and cases of babesiosis have been reported worldwide in animals and humans. Many of these species infect cattle, including *B. bovis*, *B. bigemina*, *B. orientalis*, *B. ovata*, *B. major*, *B. occultans*, *B. divergens*, *B. jakiwivi*, and *B. venatorum* [3]. In addition, BB can also be caused by *B*. U sp. Kashi, a new species identified in cattle from China [4]. Despite the scarcity of information, it is estimated that BB has a major economic impact on milk and meat cattle industries in China, causing significant losses every year. This review is aimed at increasing awareness about the current state of BB in China, the complex lacework of hosts, vectors, and pathogens, and highlight potential risks and knowledge gaps that limit progress towards control of this disease.

## Cattle, water buffalo, and yaks in China

China is one of the largest agricultural countries in the world and one of the largest markets for protein consumption. According to the United States Department of Agriculture (USDA) Foreign Agricultural Service, China is the largest beef producing country in Asia and the fourth largest in the world, behind only the USA, Brazil, and the European Union. Despite the market size and increased demand, the total cattle population in China has decreased over 40% from 2000 to 2017, while the water buffalo (*Bubalus bubalis*) population has remained relatively stable since 2012 (http://www.fao.org/faostat/en/#data/QC). By the end of 2018 the estimated inventory of cattle (not including water buffalo) in the country was around 89.2 million (http://www.stats.gov.cn/tjsj), including 10.4 million dairy cattle (China Dairy Yearbook, 2018) and 66.2 million beef cattle (National Beef Cattle Improvement Center, 2020; https://nbcic.nwsuaf.edu.cn/cyzx/76c7c38da3034eac8c4df79759bbec0b.htm). The overall decrease in cattle population from 2000 to 2017 may be due to mechanization of agricultural processes, resulting in a sharp drop in the use of animals. However, beef and milk production in 2019 were 6.67 million and 32.01 million tons, respectively (http://www.stats.gov.cn/tjsj/). Considering the population of 1.4 billion people in China, the per capita beef and milk production was only 4.766 kg/person and 22.865 kg/person in 2019, respectively. This implies immediate potential for these industry sectors to grow, especially to meet the demand as the Chinese economy and population continues to increase.

The dairy cattle population is mainly distributed in northern and eastern China and relatively less in the southern and western areas. Beef cattle are mainly concentrated in the intensive cropping areas along the central plains of Shandong and Henan provinces and in the northeast provinces of Jilin and Liaoning provinces. There are low densities of cattle in some southeastern provinces while in the more extensive grazing systems of the northwest large cattle herds are distributed over large distances. Beef cattle populations in the southwest cover diverse areas of intensive crop-cattle and grazing systems in mountainous areas.

It is recorded that 97.4% of the world water buffalo population is currently located in Asian countries and is the second largest resource of milk-yielding livestock in the world, contributing approximately 50% of Asian milk production [5]. The total number of water buffalo in China is approximately 27.1 million, which is the third numerous in the world, behind only India (114.2 million) and Pakistan (38.8 million) (FAOSTAT, 2018, http://www.fao.org/faostat/en/). The total stock of water buffalo is gradually increasing in recent decades due to economic development and the launch of water buffalo milk and meat industries in central and southern regions of China.

White yak is a rare and unique yak breed that only lives in Tianzhu Tibetan Autonomous County, Gansu province, China. These animals are an important source of food and hides, mainly for people who live in these areas. White yaks are raised in cold and harsh conditions, as the altitude of Tianzhu ranges from 2040 to 4874 m above sea level. The animals feed on natural pasture usually at the altitude of 3400 m above sea level with annual average temperature of 0.2–1.3 °C. The total population of domestic yaks is estimated to be around 14 million in China [6] while the number of white yaks is only around 39,400 [7]. White yaks have a strong ability to adapt to ecological environments with low oxygen and are indispensable multipurpose livestock for people living in Tianzhu, as these animals supply meat, dairy products, and hides [8, 9]. The complex interactions between water buffalo, white yaks, and additional breeds of bovids with *Babesia* spp. needs to be considered in order to better estimate the impact of BB on the milk and meat industry in China, and to design strategies for disease control.

In addition, the presence of native bovid species, such as yaks (*Bos mutus* and *Bos grunniens* for wild and domestic yaks, respectively), may play an important role in the dynamics of BB in China. A recent study showed that yaks might be at high risk for infestation with ticks competent for the transmission of *Babesia* spp. It has also been reported that *B. bovis*, *B. bigemina*, *B. ovata*, and *B. venatorum* are spreading in the white yak populations, which poses an additional threat to this already endangered bovine species [3, 9]. Also, climate changes may broaden the habitat of ticks competent to transmit *Babesia* spp. therefore affecting the indigenous populations of white yaks.

## Diversity of *Babesia* spp. infecting bovids in China: historical, climatic and geographic perspectives

The earliest recorded case of a disease caused by intraerythrocytic protozoa affecting cattle in China may have occurred more than 100 years ago in Chifeng city, Inner Mongolia [10]. However, officially, the first case of BB was reported in 1974 with a *B. bigemina* outbreak in Fuzhou city, Fujian province [10]. Later in the same year, 10 of 20 cows died of piroplasmosis in Jinhua city, Zhejiang province. Around the same year, 90 cows imported from New Zealand were rapidly infested with ticks, and, as a result, 15 of them died with signs of BB within 10 days after exposure to the ticks [10]. After that episode, sporadic cases of BB were reported in 19 provinces between 1949 and 1989 [10]. It was also during this period that research on babesiosis was launched in China.

**Fig. 1:**
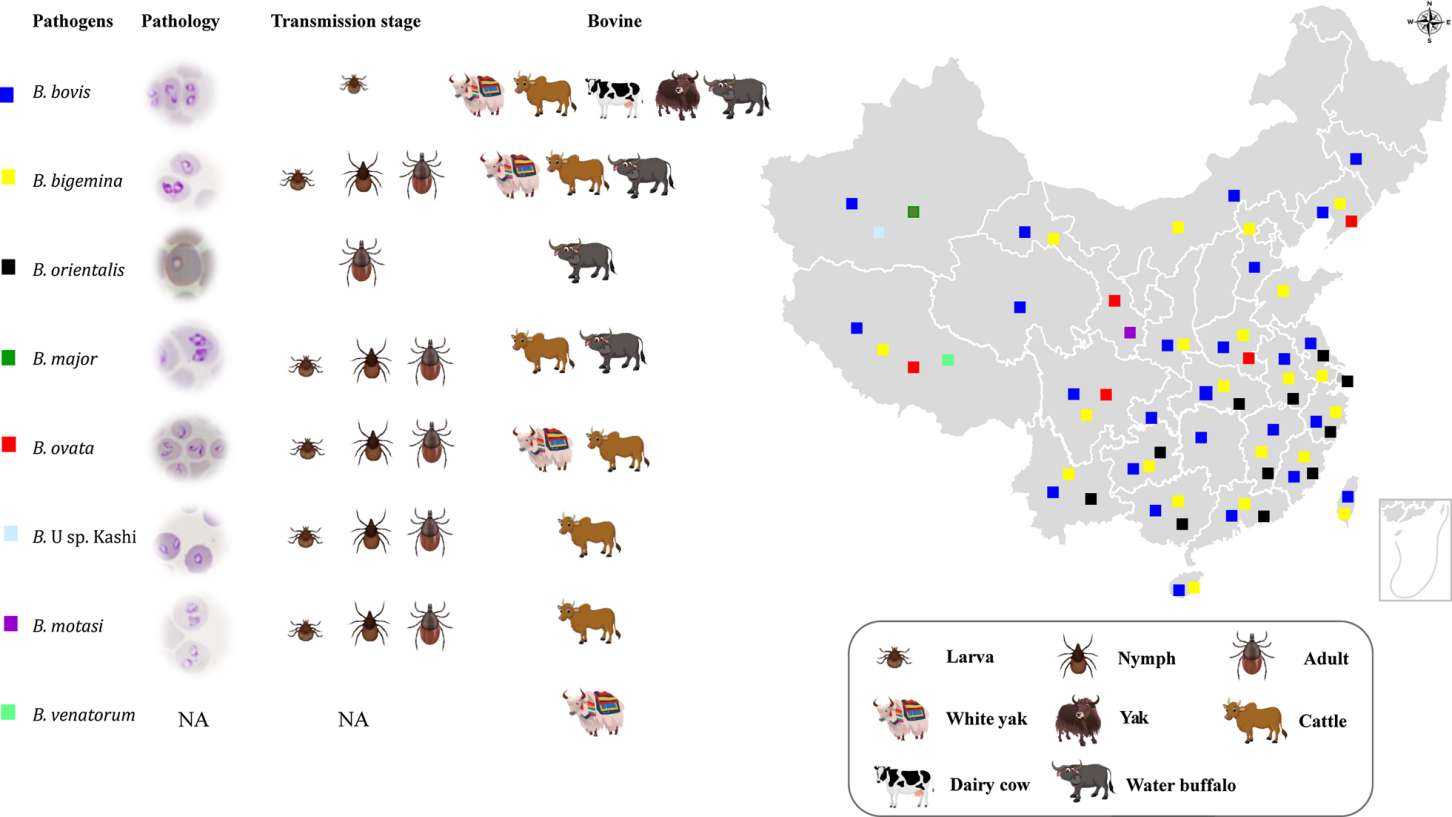
Geographical distribution of *Babesia* species detected in bovines in China. *Babesia bovis* (blue square) and *B. bigemina* (yellow square) are the most widely distributed species, following *B. orientalis* (black square) mostly endemic to the south of Yangtze river; the endemic areas of other species including *B. major* (dark green square), *B. ovata* (red square), *B.* U sp. Kashi (light blue square), *B. motasi* (purple square), and *B. venatorum* (green square) seem limited according to the present data, which may be due to the lack of widespread sampling and detection. The pathology, tick vector transmission stage, and type of hosts are shown. NA indicates not available

**Fig. 2:**
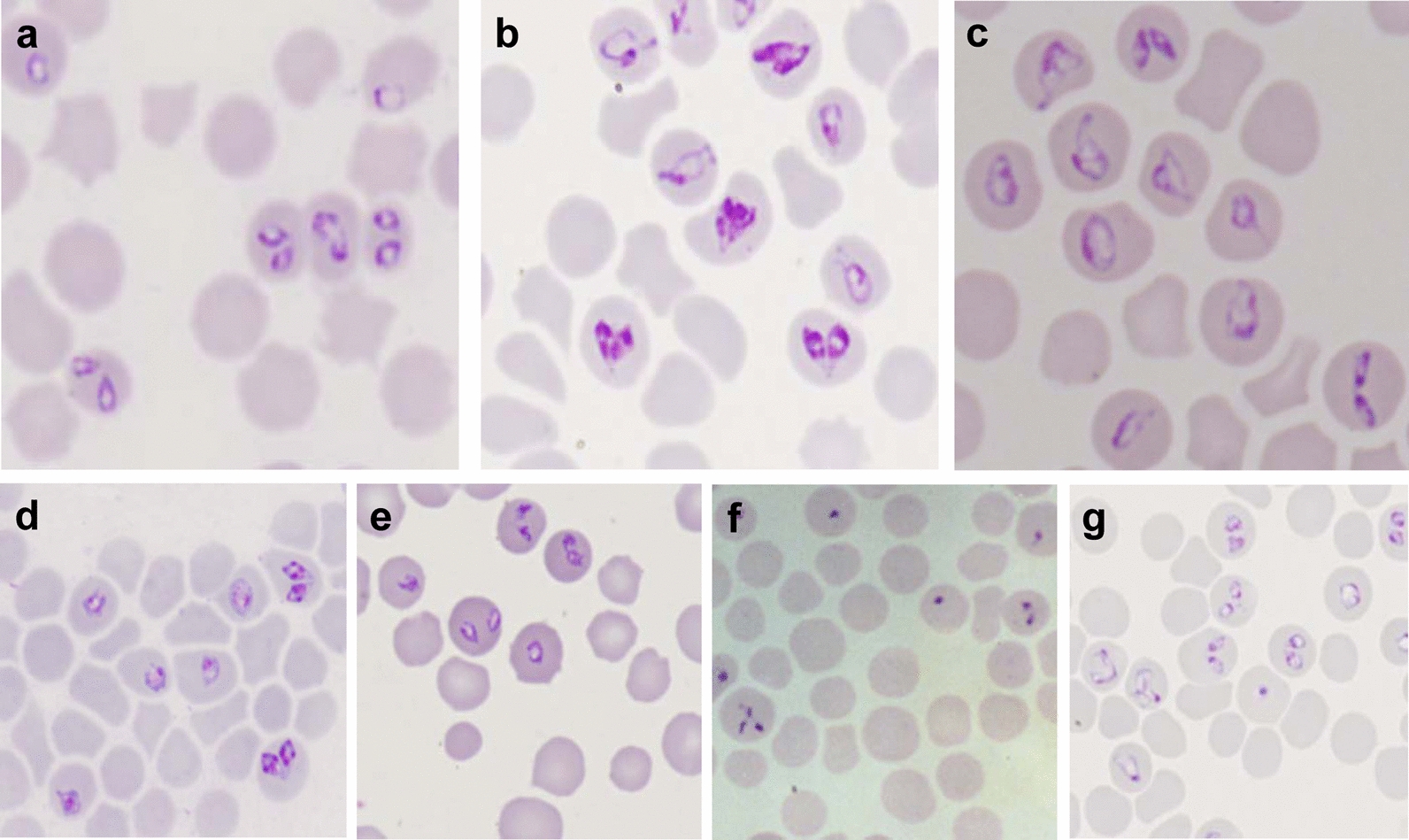
Giemsa-stained thin blood smear of bovine *Babesia* spp. Intraerythrocytic forms of *B. bovis* (**a**), *B. bigemina* (**b**), *B. ovata* (Wenchuan isolate) (**c**), *B. major* (Yili isolate) (**d**), and *B.* U sp. Kashi (**e**) were obtained from experimentally splenectomized cattle. Intraerythrocytic forms of *B. orientalis* (**f**) were obtained from an experimentally splenectomized water buffalo and *B. motasi* (**g**) from an experimentally infected sheep. Magnification ×1000

The geographical distribution of the multiple *Babesia* species detected in bovids in China is shown in Fig. 1, according to the epidemic data from 1947 up to the present day. The most widespread species in both cattle and water buffalo are *B. bovis* and *B. bigemina* [10–15] (Figs. 1 and 2). These parasites were first isolated and identified by Yang and Wang in 1964 in Guizhou province [10]. By that time, *Rhipicephalus microplus* was confirmed as the tick vector [16]. *Babesia bovis* and *B. bigemina* often co-infect cattle since they share *R. microplus* as their tick vector. Incidence of *B. bigemina*, which has been detected in 24 provinces and autonomous regions, is dependent on the distribution of *R. microplus*. However, *B. bovis* has only been reported in 21 provinces and autonomous regions [17]*.* This discrepancy may be due to the differences in the life cycle of *B. bigemina* and *B. bovis*. While *B. bigemina* can be transmitted by *R. microplus* larvae, nymphs, and adults, *B. bovis* is only transmitted by the tick larval stage [16]. In addition, the range of ticks that transmit *B. bigemina* is larger than those that transmit *B. bovis* [18]; however, in China, so far, the only competent tick species for *B. bigemina* identified is *R. microplus* [16]. Epidemiological studies have shown that the infection rates of *B. bigemina* and *B. bovis* are as high as 90% in some locations, especially in Hunan, Hubei, Yunnan, Guizhou, and Sichuan provinces [19]. An outbreak of *B. bigemina* was reported in yaks in 1989, where 309 animals died in the Sichuan province. The presence of *R. microplus* on the infected animals suggested the participation of this tick species in the outbreak [12, 13]. Recent reports also identified *B. bigemina* [9] and *B. bovis* [15] infection in yaks and white yaks in the Gansu province, which poses an additional threat to this important bovine breed [3]. In contrast, *B. orientalis*, a relatively benign species that is present in central and south regions of China, has only been described in water buffalo, and apparently does not infect cattle or yaks [20]. *Babesia orientalis* is a small parasite (Fig. 2) that has currently been reported in 12 provinces in central and southern parts of China [20]. It was first reported in 1984 in water buffalo [20–22], and subsequently demonstrated that the parasite can only be transovarially transmitted by the adult stage of the three-host tick *Rhipicephalus haemaphysaloides* [23]. Earlier studies have mistakenly identified *B. orientalis* as *B. bovis*, and/or *B. bigemina* according to the pathogen's morphology inside erythrocytes [24]. After careful examination, the parasite was renamed as *B. orientalis* in 1997 according to the differences in host susceptibility, transmission vector competence, morphology, pathogenicity, and characterization of in vitro culture [20, 25, 26]. *Babesia orientalis* can only infect water buffalo, while in contrast, *B. bovis* and *B. bigemina* can infect a diversity of bovids, including *Bos taurus*, *Bos indicus*, *Bos mutus*, and *Bubalus babalis*. In addition, *R. microplus*, a competent vector for *B. bovis* and *B. bigemina*, is unable to transmit *B. orientalis* [27]. According to epidemiological investigations, *B. orientalis* was mainly restricted to the area located south of the Yangtze River but then it started spreading to the northern area of the river in 2009. This was probably due to the construction of several bridges and the transportation of water buffalo to both sides of the river [28].

*Babesia major* and *B. ovata* were initially isolated in Xinjiang and Henan provinces, respectively [29, 30]. These two *Babesia* species caused an academic argument about species identification and validation in China. In 1988, a *Babesia* species isolated from *Haemaphysalis longicornis* ticks collected in Lushi County, Henan Province was suspected to be *B. ovata* or *B. major* [29–31]. Later, the parasite was identified as *B. ovata* [29, 31]. In order to investigate the distribution of this parasite, several isolates were obtained from different geographic regions, such as Zhangjiachuan (*B. ovata* Zhangjiachuan) and Ningxian (*B. ovata* Ningxian) county of Gansu and Wenchuan [*B. ovata* (Wenchuan isolate)] county of Sichuan province (Fig. 2) [32–34]. At the same time, an unidentified *Babesia* species was isolated from calves infested with adult *Haemaphysalis punctata* ticks collected from Yili county of Xinjiang Uygur Autonomous Region (Fig. 2). The parasite was initially characterized as *B. major*, but then renamed as *B. major* (Yili isolate) due to its vector tick [35]. Traditionally, discrimination between *B. major* and *B. ovata* is mainly based on the tick vectors involved [36, 37], considering that *B. major* is transmitted by *H. punctata* [38] while *B. ovata* is transmitted by *H. longicornis* [39]. Subsequently, Yin et al. [40] reported that *B. major* could be transmitted by both *H. punctata* and *H. longicornis* at larval, nymphal, and adult stages in experimental conditions. After that, the argument focused on whether these *Babesia* species should be classified into *B. major* or *B. ovata* considering that both species share many similarities in morphology and pathogenicity [29]. Recent phylogenetic analysis of 18S rRNA gene and internal transcribed spacer region demonstrated that *B. ovata* Lushi, *B. ovata* Ningxian, *B. ovata* (Wenchuan isolate), and *B. ovata* Zhangjiachuan, which are all transmitted by *H. longicornis*, fall into one single clade, while *B. major* (Yili isolate), which is transmitted by *H. punctata*, falls into a separate clade [34, 41]. Both these *Babesia* species have low pathogenicity in intact calves, do not cause apparent clinical disease, and are not generally considered important pathogens, in comparison to *B. bovis* and *B. bigemina* [30, 31, 40]. Correlation with *B. major* and its vector tick only prevails in the northern regions of Xinjiang Uygur Autonomous Region while *B. ovata* is possibly widespread in China due to its ability to be transmitted by *H. longiconrnis* larvae, nymphs, and adults, which are distributed in most Chinese regions [42]. *Babesia ovata* was also recently identified in white yaks in the Gansu province [3].

*Babesia* U sp. Kashi is another recently identified species found to infect cattle in China (Fig. 2). In 2002, Luo et al. described an unidentified *Babesia* species that showed low virulence in calves and which was isolated from *Hyalomma anatolicum* collected from Kashi County of Xinjiang province. This newly isolated species was designated *Babesia* U sp. Kashi [4] and subsequent experiments showed that the parasite was transmitted by larval, nymphal, or adult stages of *Hy. anatolicum*, *Hy. detritum*, and *Hy. rufipes* Koch. Interestingly, *Babesia* U sp. Kashi was not transmitted by either *R. microplus* or *H. longicornis* [43]. Molecular phylogeny using the 18S rRNA genes showed that *Babesia* U sp. Kashi is different from other *Babesia* species previously identified [44]. Additional genetic and molecular studies are needed to validate the *Babesia* U sp. Kashi as a new species and examine potential relatedness of this species with *Babesia beliceri* from Russia and *B. occultans* from South Africa, which are also transmitted by *Hyalomma* ticks [44–46].

*Babesia motasi*, which primarily infects sheep and goats (Fig. 2), was recently identified in cattle in the Gansu province, China [11]. In the 1980s, Chen [47] and Zhao et al. [48] first reported cases of ovine babesiosis in the Sichuan and Heilongjiang provinces, respectively. Considering that sheep were involved in these cases, the causative agent was mistakenly assumed to be *B. ovis*. In 1996, an outbreak of ovine babesiosis was reported in several herds of small-tailed han sheep imported from the Shandong province in Ningxian county of Gansu province. At the time, two ovine *Babesia* strains were isolated: a large *Babesia* highly virulent for sheep and goats, which was primarily designated as *Babesia* sp. or *B. motasi*, and *Babesia* sp. BQ1 (Ningxian), and a small *Babesia* with low virulence that was classified as *B. ovis* [49, 50]. In addition, it was demonstrated that the larger form of the parasite was able to be transovarially transmitted by *H. longicornis* ticks [51]. To date, a total of six *B. motasi*-like isolates have been reported in sheep and goats, including *Babesia* sp. BQ1 (Lintan), *Babesia* sp. BQ1 (Ningxian), *Babesia* sp. (Liaoning), *Babesia* sp. (Tianzhu), *Babesia* sp. (Madang), and *Babesia* sp. (Hebei) [52, 53]. Some of these isolates, such as *Babesia* sp. BQ1 (Lintan), were able to be experimentally transmitted by both *H. longicornis* and *H. qinghaiensis* at larval, nymph, and adult stages [54, 55]. In 2020, Sun et al. described the first report of *B. motasi* in cattle, showing 10% positive infection rate in Minqin city, Gansu province, China [11]. Unfortunately, at the time, no ticks were collected from infected cattle in that area. Considering the identification of *B. motasi*-like parasites in both cattle and small ruminants, it would be interesting to investigate the genetic homology of these isolates. Collectively, these results indicate that further studies are required to find out potential risks imposed by *B. motasi* to livestock in these affected areas in China.

*Babesia venatorum* (formerly *Babesia* sp. EU1) is a zoonotic parasite first reported in Italy and Austria in two splenectomized men [56], and also in deer and sheep [46, 57]. In 2014, Sun et al., reported the first *B. venatorum* case in China. An 8-year-old boy who lived in Pishan County, Xinjiang Autonomous Region was detected with a parasitemia of 5% [58]. So far, 48 human cases have been reported in China [59], and have later been identified in white yaks from Tianzhu Tibetan Autonomous county, China, in 2017 [3]. A total of 409 samples were tested by nested PCR targeting the 18S rRNA gene. Four samples were positive for *B. venatorum* based on the sequences. No tick vectors were collected from the infected white yaks; however, *Ixodes ovatus* ticks were collected from Tianzhu Tibetan Autonomous County. Vectors implicated in the transmission of *B. venatorum* are currently unknown. However, *Ixodes persulcatus* has been suggested as a competent vector for *B. venatorum* in Mongolia [60] and Heilongjiang province, China [59]; however, additional investigation is needed to test this hypothesis.

*Babesia divergens* is a well-known zoonotic species that infects bovines. This parasite can be transmitted by *I. persulcatus*, which is widespread in China. However, interestingly, *B. divergens* infections have been reported in humans and ovines, but not in bovines in China. Qi et al. [61] reported in 2011 the first *B. divergens*-infected human case in Shandong province, China. A total of 377 human blood samples were analyzed and two were found positive for *B. divergens* 18S rRNA, as confirmed by sequencing. Wang et al. [62] demonstrated a 1.3% (10/754) positive rate tested by 18S rRNA PCR in human samples in the Gansu province, China. *Ixodes persulcatus* is a competent vector for *B. divergens* that is widespread in China [63]. Despite the concern of *B. divergens* as a zoonotic pathogen and that all cases involved humans, no data are available of the incidence of this parasite in bovines. Further studies are needed to investigate additional competent vectors and the impact of *B. divergens* in bovines from China, especially considering that *I. ovatus* is also a possible competent vector for this parasite in infected dogs from Japan [64]. Also, these investigations can potentially bring to light the implication of *B. divergens* for both human and animal health in China.

*Babesia occultans* is considered a benign species that primarily infects cattle and it was first described in South Africa in 1981 [46]. The first suspected report of *B. occultans* in China was in 2018, and also at that time, parasites were identified in *Dermacentor nuttalli* collected from sheep in Xinjiang Uygur Autonomous Region, China [65]. Unfortunately, blood samples of the infected sheep were not collected and tested, therefore, there was no confirmatory evidence of the presence of *B. occultans* in sheep in these areas. In 2019, Sun et al. [66] reported *B. occultans* in *Hyalomma asiaticum* collected from sheep in the Gansu province, China. *Babesia occultans* was speculated to be originally a parasite typically infecting African antelope, which can explain the presence of the parasite in ticks collected from co-grazing ovine. Although potential competent vectors are widespread in China, *B. occultans* infection has not been identified in bovines and further epidemiological, genetic, and pathogenicity studies need to be performed on *B. occultans* in bovines to understand its prevalence in China.

Importantly, *I. persulcatus*, which is a competent vector for *B. divergens*, as well as *D. nuttalli* and *Hy. asiaticum* ticks, which are also vectors for *B. occultans*, are also widespread in China (Table 1). Considering this scenario, it is likely that both, *B. divergens* and *B. occultans* are already circulating among cattle in the country, posing additional risk for the cattle industry and human health. Therefore, confirmatory epidemiological studies should be conducted to evaluate the actual impact of *B. divergens* and *B. occultans* in bovines in the Chinese provinces.

**Table 1:** *Babesia* species infect cattle and water buffalo in China

Species	Size (µm)	Pathogenicity	Host	Tick vector	References
*B. bovis*	2.0 × 1.5	Severe	Cattle, water buffalo, yak, white yak	*R. microplus, R. annulatus* (N), *R. geigyi* (N)	[3, 10, 16, 101]
*B. bigemina*	4.5 × 2.0	Moderately severe	Cattle, water buffalo, white yak	*R. microplus, R. decoloratus* (N)*, R. annulatus* (N), *R. geigyi* (N), *R. evertsi* (N)	[3, 9, 10, 16, 102]
*B. orientalis*	1.2–1.5 × 2.0–2.6	Severe	Water buffalo	*R. haemaphysaloides*	[20, 22, 23, 26]
*B. ovata*	3.2 × 1.7	Benign	Cattle, white yak	*H. longicornis*	[3, 29, 31]
*B. major*	2.6–3.7 × 1.5	Benign	Cattle, water buffalo	*H. punctata*	[34, 41]
*B*. U sp. Kashi	1.5–5.0 × 1.2–3.0	Benign	Cattle	*Hy. anatolicum*	[4, 44]
*B. motasi*	2.5–4.5 long	Moderately severe	Ovine cattle	*H. punctata, R. bursa*	[11, 47, 48]
*B. venatorum*	Unknown	Unknown	White yak	*I. persulcatus*, *I. ovatus*	[3]
*B. divergens*^a^	1.5–2 × 0.4	Moderately severe	Human, ovine	*I. ricinus* (N), *I. persulcatus*	[61, 62]
*B. occultans*^a^	2.9 × 1.2	Benign	No report in host, but detected in ticks in China	*D. nuttalli, Hy. asiaticum*	[65, 66]

N: not reported in China; *B*: *Babesia*; R: *Rhipicephalus*; *H*: *Haemaphysalis*; *Hy*: *Hyalomma*; *I*: *Ixodes*; *D*: *Dermacentor*

^a^*B. divergens* and *B. occultans* have not been reported infecting cattle in China

As can be appreciated from the presented data, there is a large diversity of *Babesia* organisms circulating among distinct ticks and bovids in China, and as more research is performed, it is likely that other unknown species may be discovered in the future. Furthermore, it is interesting that some parasite species that are known to specifically infect certain hosts, such as *B. motasi*, a parasite that causes disease in sheep, were only identified infecting cattle in China [11]. This situation differs dramatically from other endemic countries, such as Australia, Brazil, Mexico, and Argentina, among other countries, where the diversity of *Babesia* spp. appears to be much more limited and usually restricted only to *B. bovis* and *B. bigemina* [67–70].

Also, the presence of native bovid species, such as yaks (*Bos mutus* and *Bos grunniens* for wild and domestic yaks, respectively), may play an important role in the dynamics of BB in China. A recent study showed that yaks might be at high risk for infestation with ticks competent for the transmission of *Babesia* spp. It has also been reported that *B. bovis*, *B. bigemina*, *B. ovata*, and *B. venatorum* are spreading in the white yak populations, posing an additional threat to this already endangered bovine species [3, 9]. Also, climate changes may broaden the habitat of ticks competent to transmit *Babesia* spp., therefore affecting the indigenous populations of white yaks.

In summary, although *B. bovis* and *B. bigemina* are the most widespread species in cattle, water buffalo, and yaks in China, followed by *B. orientalis*, *B. major*, *B. ovata*, and *B.* U sp. Kashi (based on the data collected in this review), there is a remarkable diversity of *Babesia* spp. and competent ticks circulating in the country. In addition, cross-species infections, such as the case of the sheep parasite *B. motasi* in cattle, suggest the presence of a complicated and unique scenario.

BB is a complex and difficult disease to control even in usual situations involving a few parasite species and their respective competent tick vectors. Circumstances in China are complex in terms of the *Babesia* species, ticks, and vertebrate hosts involved, and need to be considered if implementation of control strategies, such as vaccination, anti-Babesia drugs, and acaricides are to be effective.

## Risk factors

Intrinsic risk factors associated with BB include animal age, nutrition and general sanitary conditions, use of vaccines and babesicidal drugs, breed of the animals, potential for infestation by tick species circulating in the study area, and the existence of preventive measures against ticks [67]. The impact of animal age is very important, given the increased resistance of young calves to acute disease [71]. The nutritional status and general well-being of the animals are obvious risk factors, with well-fed animals having an advantage and better chances to survive the impact of acute BB than emaciated and malnourished cattle [67]. Cattle breed is also considered a risk factor, since *Bos taurus* breeds are remarkably more susceptible to *Babesia* infections compared to *Bos indicus* cattle [72, 73]. The latter species also have increased resistance to tick infestation. This is likely the result of long-term selection due to the evolutionary history of the native *Bos indicus* breeds that co-evolved with ticks and tick-borne parasites. Management practices play an important role, and systems that favor close proximity of animals may pose increased risk to tick infestation and tick-borne diseases. On the other hand, extrinsic factors that may increase the severity of the situation include other and often uncontrollable players, such as climate, animal movement, and human activities [28]. Together, worldwide climate change and globalization-related practices have resulted in a fast expansion of favorable habitat for the development of ticks and tick-borne diseases, such as BB. In addition, increased industrialization, human encroachment, and invasion of natural habitats have resulted in displacement of wild and domestic animals. It is predicted that all these risk factors mentioned above may play a role in BB in China and represent potential areas where more research is needed to develop efficient strategies to control the disease.

## Critical knowledge gaps

Considering the increased demand for animal protein and that BB is currently a potential threat to the cattle industry in China [17], as it is for *Babesia*-endemic countries in Africa, the Americas, and Australia [18, 67, 74–77], it is critical to have a plan to evaluate impact and to control the disease. To this end, it is necessary to fill critical knowledge gaps and find appropriate answers to some important questions related to this economically important disease. These include gaps in the dynamics of BB in China, whether the risks for cattle populations are increasing or decreasing in the country, whether the disease is confined to specific location or provinces, or whether it has the potential to expand geographically, among many regions, and the impact of possible wildlife reservoirs. Importantly, regions in the country with enzootic stability versus instability need to be assessed. Considering that only a few studies have addressed these issues so far, the overall impact of BB in China is currently unknown. Important issues that need to be considered in this assessment include understanding how environmental changes and human interventions are affecting populations of *Babesia*-competent tick vectors, and what measures would be required to mitigate the effect of *Babesia* on the production of cattle milk and meat in China. A complete survey on the *Babesia* spp. that infect and cause clinical disease in China should be performed. This includes the recently identified *Babesia* sp. Mymensingh that can cause clinical babesiosis and was found endemic for cattle and water buffalo in Asia, Africa and South America. The presence of this and other *Babesia* species of cattle and buffalo in China should be also investigated [78]. Chief among such possible measures is the design of a proper, environmentally safe, and efficient strategy aimed at controlling ticks using acaricides, developing or improving current anti-tick vaccines [79–81], or pasture management, among other possibilities [82].

## Prospective research needs

As successfully demonstrated by the tick control campaign in the US, which extended for about four decades [83, 84], control of babesiosis can be achieved by targeting ticks with acaricides [83]. However, this success was not reproduced elsewhere, and the extensive use of acaricides may be detrimental to the environment. Other environmentally-friendly measures for tick control, such as anti-tick vaccines and natural repellents, could also be considered as sustainable options [85–90]. Control of BB is a difficult task due to the paucity of efficient drugs and vaccines, and also to the fact that the causative agent is diverse and widespread in the environment. Regarding BB research in China from the last decades, numerous studies have been performed, and consequently new information on the life cycle of different parasite species isolated from different stages of tick vectors has been revealed [16, 26, 30]. Importantly, major progress has also been made recently in the establishment of in vitro cultivation of *Babesia* parasites, epidemiology surveys based on molecular and serological diagnostic methods, and screening of diagnostic and vaccine antigens [11, 15, 20, 91, 92]. Despite these advances, no commercial diagnostic kit or vaccines are currently available in China. In most endemic areas in the world, immunologically naïve imported animals usually succumb to acute babesiosis [93]. Live vaccines, based on native *Babesia* strains, may be useful to protect imported *Babesia* naïve cattle that could be used to improve productivity in China. In addition, in the absence of alternative preventive strategies, live vaccines combined with diagnostic surveys may contribute to alleviating the burden of disease in endemic areas whenever they become enzootically unstable [81, 94].

It is well-accepted that BB causes enormous economic loss in endemic areas [95]; however, the exact global economic impact remains unknown, and this is especially true in China. Thus, as a first step to address the situation, commercial diagnostic kits are urgently needed to perform epidemic surveys of babesiosis in the bovid populations of China. Such surveys are essential to inform whether a certain region is endemic or free of *Babesia* spp. Diagnostic surveys will also determine if enzootically stable and unstable regions are present in the country [96, 97]. Importantly, once enough epidemiological information is available, design of control plans may be aided by developing predictive models which may address the potential for expansion of the tick vectors into endangered areas [98]. For instance, epidemiological surveys could indicate the need for vaccination in enzootically unstable regions in China, by increasing the numbers of *Babesia*-immune animals in at-risk herds. However, exposure of cattle to the parasite in *Babesia* endemic areas should ideally occur at an early age (< 1-year-old), since younger animals are highly resistant to the infection and develop only mild signs of acute babesiosis. In contrast, older naïve animals (> 1-year-old) usually succumb to acute infection. In addition, this also applies to live vaccines based on attenuated parasites, which can only be administered to young animals (< 1-year-old).

As mentioned, *B. bovis* and *B. bigemina* are the most virulent and prevalent species in endemic regions worldwide. While *B. bovis* causes more dramatic acute disease due to its ability to sequester in several tissues, including brain vascular epithelia causing cerebral babesiosis [94], *B. bigemina* is characterized by acute hemolytic anemia that may result in kidney damage and organ failure. The relative virulence of additional *Babesia* spp. described above remains uncertain and needs to be investigated. In addition, the presence of *Babesia* parasites, albeit of low virulence, may pose an important risk when animals undergo stress, immunosuppression, or co-infections with other agents. In these circumstances, clinical disease leading to death may manifest. Finally, if these “lowly virulent” parasites cause persistent infection and remain in circulation, they can be transmitted by competent ticks to other susceptible animals. It may be important to determine whether these alternative and poorly virulent parasites are present in China and whether control measures are needed.

There is a clear need to screen and identify novel *Babesia* targets to design and develop effective drugs for endemic areas worldwide, including China. Diminazene aceturate and imidocarb dipropionate are usually recommended and are very useful drugs for babesiosis, but they may not fully eliminate the parasites from the infected host [95]. Therefore, new vaccine strategies are also required for the implementation of efficient strategies to control and eradicate *Babesia* spp. A possibility is the use of gene manipulation in cultured attenuated parasites to develop better defined and non-transmissible live vaccines [67, 94]. Another strategy is to develop subunit vaccines based on multiple essential antigens including both asexual and sexual stages of the parasite [94]. Future studies should focus on parasite-tick-host interactions, parasite metabolisms, and potential reasons that *Babesia* can only survive inside erythrocytes. It has been well established that erythrocytes lack immune receptors, such as MHC, and provide a safe environment for the parasites to evade the vertebrate host immune system [99, 100]. Understanding the mechanisms of erythrocyte invasion and parasite biology inside of erythrocytes should reveal new ideas for controlling the disease, allowing the design of novel pan-babesicidal drugs and effective vaccines. Collectively, by addressing these gaps and considering all the complex cultural factors present in China, including the consumption of specific beef-based food and the predicted increase demand of animals that follows economic growth, among other aspects, efficient strategies to evaluate the impact of BB in the country would be an advantage to cattle production.

## Conclusions

BB is a significant limiting and neglected factor for the development of the cattle industry in China. There is still much to be learned on parasite diversity and the dynamics of the disease in the country before methods of control can be applied effectively. One first step is the development of large-scale surveys and the identification of possible natural reservoirs that can play a role in the dynamics and expansion of the disease in the country. Development of efficient, simple diagnostic tools are prerequisite steps required for relevant epidemiological survey studies. Also, live attenuated vaccines based on local parasite isolates may be required to control outbreaks in enzootically unstable regions. Additionally, subunit vaccines that are shown to be efficacious against circulating *Babesia* species and isolates might be needed. These will require a better understanding of the biology of the parasite, its interactions with vertebrate and arthropod hosts, and the interactions occurring among farm and wild animals that may act as reservoirs. Other factors that directly or indirectly affect the manifestation of the disease may also need to be better understood, such as environmental and climate factors. Geographic and epidemiological data can also be used for developing predictive models for the expansion of the vector ticks [98]. More investments in basic and applied research of the disease are urgently required to attack this problem rationally. As for the current impact of BB in China, it may be necessary to bring together teams of government officials, economists, scientists, cattle industry stakeholders, and other important actors, to develop a comprehensive epidemiological analysis, followed by the design of comprehensive strategies against BB in China. This approach can potentially reveal the full impact of the disease and provide an efficient platform to deliver stable and reliable sources of food to provide for the needs of an increasing and more demanding Chinese and worldwide population.

## Data Availability

Not applicable.

## Abbreviations

BB: Bovine babesiosis
*R. microplus*: *Rhipicephalus microplus*
*R. **haemaphysaloides*: *Rhipicephalus* *haemaphysaloides*
*H. longicornis*: *Haemaphysalis longicornis*
*H. punctata*: *Haemaphysalis punctata*
*Hy. anatolicum*: *Hyalomma anatolicum*
*Hy. asiaticum*: *Hyalomma* *asiaticum*
*Hy. detritum*: *Hyalomma detritum*
*Hy. rufipes*: *Hyalomma rufipes*
*I. persulcatus*: *Ixodes persulcatus*
*I. ovatus*: *Ixodes ovatus*
*D. nuttalli*: *Dermacentor nuttalli*
